# A quasi-experimental study of community-based financial capability intervention and depression in Central Mozambique

**DOI:** 10.1007/s44192-026-00492-7

**Published:** 2026-08-11

**Authors:** Aweke Tadesse, Michael G. Vaughn, Mintesnot Teni, Julie Birkenmaier, Jesse J. Helton, Armando Licoze, Jin Huang

**Affiliations:** 1 School of Social Work, Fairmount College of Liberal Arts and Sciences, Wichita State University, Wichita, KS USA; 2 School of Social Work, Saint Louis University, St. Louis, MO USA; 3 College for Public Health and Social Justice, Saint Louis University, Wool Center, St. Louis, MO USA; 4 Oasis Mozambique, Beira, Mozambique; 5 Brown School of Social Work, Washington University in St. Louis,, St. Louis, MO USA

**Keywords:** Financial capability, Village savings and loan groups, Depression, Household assets, Neighborhood social capital, General health, Household hunger

## Abstract

Poverty imposes multiple stressors that heighten mental health challenges, especially among low-income women. Community-based financial capability (FC) interventions have the potential to improve mental health while strengthening household assets, food security, social support, and general health. This study analyzed de-identified data from a February 2023 program evaluation in Central Mozambique, using a post-test-only quasi-experimental design with a random subsample of 653 women (317 intervention, 336 comparison). Multiple linear regression and Structural Equation Modeling (SEM) were used to assess the influence of FC participation and duration on depression, with household assets, neighborhood social capital (NSC), general health, and hunger as mediators. Results showed that FC participants had significantly lower depression scores than non-participants. SEM analyses indicated inverse associations between participation (β = − 0.78, ov*p* < .05) and duration (β = − 0.30, *p* < .01) with depression. Participation was positively associated with household assets and general health and negatively with household hunger. Mediators revealed that NSC and household hunger were positively linked to depression, while general health had a strong inverse link. FC participation influenced depression both directly and indirectly through general health (β = − 0.08, *p* < .01) and household hunger (β = − 0.19, *p* < .01), and duration showed indirect pathway via hunger (β = 0.04, *p* < .05). The SEM models explained 51% (R^2^ = 0.51) and 52% (R^2^ = 0.52) of the variance in depression. These findings suggest that FC interventions are scalable, community-driven strategies that reduce the probability of psychological distress and improve life outcomes in resource-limited settings.

## Background

Poverty and its cascading effects remain pervasive worldwide, often deepening survival crises and intensifying mental health conditions such as depression [1]. The World Health Organization [2, 3] has warned that without adequate intervention, depression could become the leading cause of disease burden by 2030. Currently, an estimated 4.4% of the global population suffers from depression, contributing to approximately 850,000 suicide-related deaths annually [3]. Between 2005 and 2015, depression rates rose by 18.4%, making it the world’s second most burdensome illness [4]. Despite its scale, treatment gaps remain substantial, especially in developing countries/low-resource settings [5].

Depression significantly undermines economic productivity through increased absenteeism, reduced workplace performance, and heightened risk of unemployment and poverty [6]. Globally, mental health costs are projected to reach $16 trillion by 2030, with depression being a major contributor [7]. In many low-resource settings worldwide, over 30% of primary healthcare visits are linked to depressive symptoms, which also erode social and occupational functioning [8]. The economic toll, even in high-resource countries, is profound, with annual economic losses estimated at $36.6 billion in the U.S. and £23.5 billion in Canada alone [9].

The challenge of depression is especially acute in Africa. A meta-analysis of 23 studies across Africa estimated a pooled depression prevalence of 43.1% among 14,350 older adults, with one in five experiencing depressions [10]. A longitudinal study from Ghana and South Africa (Wave 1, 2007–2008) involving 4209 Ghanaian and 3148 South African adults aged 50 and above reported 12-month depression prevalence rates of 7% and 4%, respectively [11]. In Mozambique, mental health challenges are closely linked to poverty and social and financial instability [12–15]. Studies in Sub-Saharan Africa (SSA) and other low-resource settings have found associations between low financial capability (FC) (i.e., one’s capacity for financial behaviors that benefit their financial well-being), mental health issues, and reduced quality of life [1, 16].

Little research provides guidance about interventions to improve financial capability, mental health, and quality of life among low-income women in SSA. This study examined the impact of an FC approach called “Village Savings and Loan Groups (VSLGs)”, a community-based FC intervention, on depression among low-income women in rural Mozambique. VSLGs are informal, community-led financial groups formed by individuals with shared geographic, social, or cultural ties [17, 18]. We hypothesized that both participation and length of involvement in VSLGs would influence levels of depression, with this effect mediated by social connections, household asset ownership, general health, and food security.

## Literature review

### Conceptual framework: financial capability

The conceptual framework for this study, FC, integrates financial knowledge, access, and use, leading to better financial behavior and improved life outcomes [19, 20]. Sherraden [21] views financial capability as the integration of financial literacy and access, which together foster financial confidence and motivation. Financial capability is a multidimensional concept that refers to individuals’ ability to access financial services, effectively manage financial resources, and achieve improved financial behaviors that in turn lead to well-being outcomes. It incorporates three interrelated components. Financial access refers to the availability and use of financial products and services, such as savings mechanisms, credit, and financial support systems. Financial behavior reflects how individuals manage financial resources, including saving, borrowing, budgeting, and investment practices. This combination enhances individuals’ skills and opportunities to improve their financial well-being. According to Sherraden’s financial capability framework, both individual behavior and social norms, particularly financial socialization, and institutional structures play a vital role. These factors are especially critical in low-income and vulnerable communities, where gaps in financial knowledge and skills are compounded by limited access to affordable and inclusive financial systems. Such systems are essential for enabling asset-building and long-term financial security [22].

Grounded in Michael Sherraden’s financial capability framework, financial inclusion is understood as the integration of financial knowledge, access, and use that promotes improved financial behaviors and well-being outcomes. This perspective aligns with Amartya Sen’s Capability Approach, further developed by Martha Nussbaum, which conceptualizes women’s empowerment as the expansion of substantive freedoms rather than solely income gains [23]. Financial inclusion enhances women’s capabilities when access to credit and microfinance strengthens agency, control over resources, and meaningful choice [24]. However, structural poverty and gendered constraints may limit women’s ability to convert financial access into genuine empowerment [25]. From a mental health perspective, well-being is also a capability, as it enables the freedom to function, participate, and live with dignity [26]. Together, these perspectives suggest that financial capability operationalizes how expanded opportunities are translated into financial behaviors and improved life outcomes.

This study also draws on Women’s Empowerment Theory (WET), which emphasizes access to resources and participation in decision-making as pathways to agency and strategic life choices [27, 28]. In rural African contexts, where cultural norms and structural barriers often limit women’s economic participation, community-based financial interventions like VSLGs may serve a dual role: they function as mechanisms for improving financial access (FA) while also producing direct financial outcomes through asset accumulation and economic stability. This framing positions VSLGs not merely as conduits to formal financial systems, but as financial structures embedded within communal and informal financial practices common in rural SSA settings.

FC approaches that center on FA strategies are linked to increased productivity, economic growth, and improved living standards. FA, a core element of FC, refers to the ability to own and use affordable and accessible financial services, such as those needed for saving, transacting, borrowing, and investing [29, 30]. Financial outcomes (i.e., VSLG) represent the results of financial engagement, including asset accumulation, improved household economic stability, food security, and psychosocial well-being. Within this framework, community-based financial interventions such as VSLGs are expected to strengthen financial capability by improving access to financial services, promoting positive financial behaviors, and contributing to improved financial and well-being outcomes. However, in rural SSA, structural barriers such as limited banking infrastructure and scarce resources hinder FA and therefore hinder the achievement of higher levels of FC [19]. FC strategies remain underutilized in Africa, where only 23% of adults have financial access, and in Mozambique, just 20% of women have access to financial services [31]. As indicated by the Global Findex data (2021), despite positive developments through mobile banking initiatives such as M-Pesa, Mkesh, and e-mola in countries like Senegal, Mali, and Mozambique, banks in Sub-Saharan Africa remain ill-equipped to serve the very poor and rural populations [32, 33]. Barriers such as a lack of proper documentation, limited infrastructure (e.g., distance to bank branches), and insufficient funds to meet minimum balance requirements persist and contribute to significant gaps in access to savings and credit services [34, 35].

### Village saving and loan group’s (VSLG) potential to increase financial access

VSLG emerged in low-resource settings to provide avenues for credit among those with little [6]access to credit from formal financial institutions [36]. VSLGs were introduced in Mozambique by CARE International in the 1990s as a rural development strategy. However, there is limited empirical information and evidence-based reports on the influence of VSLGs in rural low-income communities in Mozambique [37]. Typically comprising 15–30 members, these groups meet regularly to pool savings and issue loans to group members, generating returns through interest and dividends [38]. A key component of VSLG dynamics is the monthly group meeting, where members formally save, borrow, repay with interest, and discuss group matters. These meetings also encourage continued participation and help members strengthen their FA skills. While the structured activities focus on financial routines, the meetings also foster informal interactions. Members share personal challenges, exchange experiences, and develop skills such as decision-making, problem-solving, and networking [39]. These interactions, rooted in culturally familiar practices of mutual support and reciprocity, promote help-seeking behaviors.

### Evidence about VSLG outcomes

**Fig. 1:**
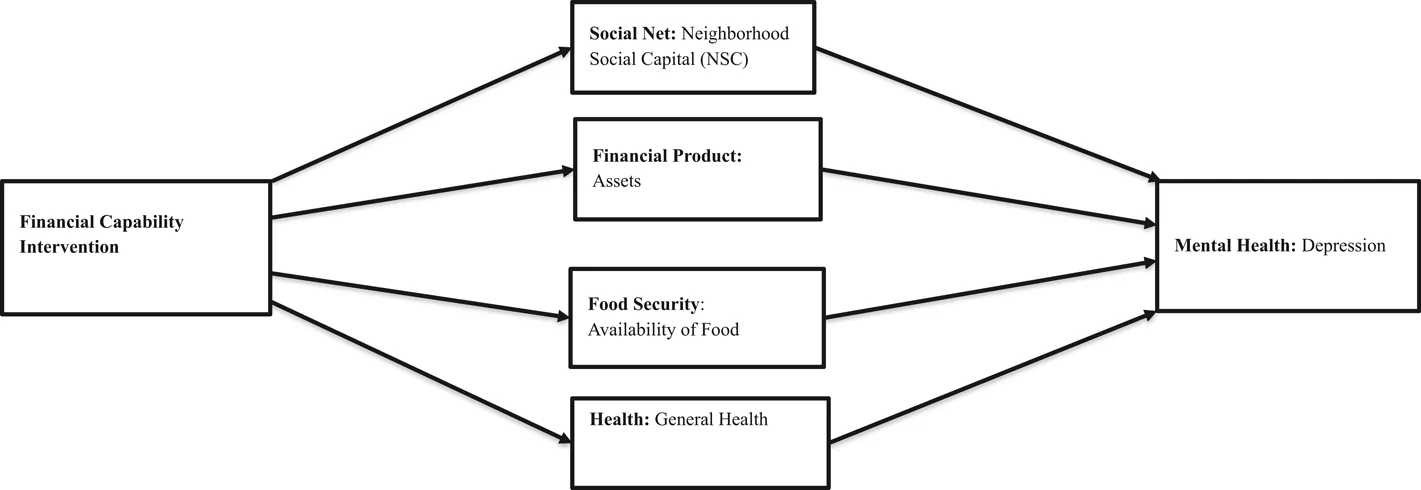
Study conceptual framework

Current evidence suggests that VSLGs have positive financial and other outcomes. Participation in VSLGs increases participants’ savings, investments, and coping capacity, helping buffer financial shocks and reduce mental health risks [40]. Improvements made from using the funds saved and accessed from VSLGs, such as addressing basic needs and better access to health service utilization, positively impact mental health and living standards [1, 5, 41, 42]. In one study in Kenya and Uganda, wealth and financial resources achieved through VSLGs, and other FC interventions were inversely associated with depression, even after controlling for demographic factors [43, 44]. Figure 1 below illustrates the study’s conceptual framework.

Evidence also provides suggestions for the mechanisms through which VSLGs have positive mental health and financial outcomes. The VSLG model fosters interpersonal skills that support emotional resilience and reduce depression risk [45, 46]. Savings behavior encourages micro-investments, asset accumulation, and income generation, all linked to mental and social well-being [41, 47]. Financial skills training through FC initiatives builds financial confidence and literacy, which are associated with better health outcomes [48–50]. Overall, VSLGs offer a scalable pathway to improving both financial and non-financial outcomes by increasing access to key resources, financial, social, and health-related, that help alleviate stressors linked to depression [18, 51].

### Depression and finances

Studies have suggested a link between limitations in (FC) (i.e., financial literacy, financial performance, and financial behavior) and financial stress, which contributes to mental illnesses such as depression and anxiety [52]). Nissa and Nissa’s study further indicated the role of FA in alleviating poverty through the mediating role of financial self-efficacy. The results suggest a positive impact of FA against financial stress, which is also linked to anxiety and depression markers. This implies that individuals with better control over financial matters have a higher probability of achieving overall health and well-being. Similarly, Demirgüç-Kunt and colleagues (2020) highlighted the influence of FA in promoting mental health outcomes. Across several studies, FA (e.g., the use of savings accounts, insurance, and digital financial tools) was associated with improved coping skills, reduced psychological stress, and enhanced overall well-being [29, 53].

## Study justification

While previous studies have explored the role of VSLGs in promoting FA and social cohesion [41, 49, 50], they often fall short in addressing the complex relationship between FC and mental health. Most focus on access and economic outcomes, such as savings behavior, credit access, and group dynamics, while overlooking the psychological and emotional impacts, especially among low-income populations and women in resource-constrained settings. This lack of integration between financial and mental health outcomes in low-resource settings with women highlights a significant gap in the literature [1].

This study aims to fill that gap by examining how VSLG participation influences both FC and mental health, particularly in low-income regions where the financial consequences of depression are most severe. The issue is especially noticeable in African contexts, where women face disproportionately low financial access and high rates of mental health challenges [6]. Drawing on the conceptual framework and previous related studies, we hypothesize that: (H1) Participation in VSLGs is inversely associated with depression; (H2) Longer duration of VSLG participation is inversely associated with depression; and (H3) These relationships are mediated by improvements in household assets, general health, and food security, and by changes in neighborhood social capital. These hypotheses frame the analysis of how community-based FC interventions influence women’s empowerment and mental health in African settings.

## Method and design

### Data collection

This study employed a post-intervention cross-sectional design using de-identified data collected in February 2023 as part of a livelihood intervention evaluation in central Mozambique. Trained enumerators and Health Science students from a local Catholic university conducted interviews under the supervision of an external researcher and non-governmental organization (NGO) staff. Questionnaires were developed in English and translated into Portuguese, which is the local language. Participants were drawn from historically impoverished rural areas of Mozambique, such as Manga Loforte, in Beira locality. Households were selected from a local government registry based on vulnerability criteria, including monthly household income under 10,000 MTZ ($160), lack of assets or employment, caregiving burdens, and lack of external support. Participants were provided with study information in the local language, and written informed consent was obtained before participation.

The VSLG implementing agency selected both the intervention and comparison groups from the same geographical area using the same selection criteria, in collaboration with the health department and community leaders. A household registry was used to identify target households based on established vulnerability criteria. Due to gender imbalance and women’s cultural and socioeconomic barriers, women were initially prioritized as the agency’s FC and empowerment targets.

For the study group, all VSLG participants were first stratified into three duration groups based on their participation in VSLG. The three durations in VSLG were defined as less than two years (n = 2846), two to four years (n = 1946), and five years and above (n = 1113), where the maximum length of duration in VSLA membership was seven years. Next, 365 individual respondents were randomly selected from the three VSLG duration membership strata, with sample sizes of 176, 120, and 69, respectively. Similarly, for the comparison group, 367 individuals were randomly selected from the non-VSLG waiting list (N = 2846). The total sample comprised 732 participants, of whom 92.9% (n = 680) were women and 7.1% (n = 52) were men. The analytic sample was restricted to female participants for three reasons. First, the small number of male participants (n = 52) was insufficient for stable gender-stratified analyses and introduced risk of biased estimates in the statistical model. Second, VSLG programs in sub-Saharan Africa are predominantly designed for and composed of women, and the mechanisms linking VSLG participation to depression, including women’s economic empowerment, social capital, and intimate partner dynamics, are theoretically and empirically distinct from those relevant to male participants[38]. Third, restricting the sample to women improves internal homogeneity and allows findings to speak directly to the population for whom this intervention is intended.

Baseline data were not used in this study because VSLG implementation was severely disrupted by major natural disasters that affected program continuity and participant stability in Sofala Province, particularly in Beira. Although the program began between 2015 and 2017, large-scale climatic disasters in 2019 caused widespread destruction of infrastructure, livelihoods, and community support systems, affecting approximately 1.85 million people and destroying more than 111,000 households [54, 55]. These disasters disproportionately affected low-income populations, especially those residing in flood-prone areas, resulting in displacement, asset loss, and interruption of program participation. As Hope and Charrua studies indicate, such large-scale humanitarian disruptions can erase or substantially alter program-related outcomes, making pre-disaster data unreliable for comparison and potentially introducing bias in longitudinal analyses.

### Ethical approval

All protocols were approved by the human subjects’ institutional board of Saint Louis University [0054746–1]. Informed consent was sought from the participants before conducting data collection.

### Measurements

#### Dependent variable

*Depression:* Depressive episodes were measured using the short version of the Edinburgh Depression Scale (EDS). The tool is widely used to assess symptoms and episodes of depression and anxiety over the past seven days. Previous studies indicate that the EDS has strong internal consistency (Cronbach’s α up to 0.88) and has been used in low-income regions [56, 57]. For this study, depression scores were measured using a 7-item questionnaire, where participants selected one of four responses that best described how they felt over the past seven days. Each depression symptom was assigned a value from 1 to 4 [1 = rarely or none of the time (0 days), 2 = some or a little of the time (1–2 days), 3 = occasionally (3–4 days), and 4 = most or all of the time (5–7 days)]. The summative score ranges from 7 to 28, with higher scores indicating more severe depressive episodes [58]. The internal consistency of the EDS in the current study sample was strong (Cronbach’s α up to 0.90).

#### Independent variables

*Participation in FC community-based intervention***:** Participation in VSLG was collected using binary responses: “Yes” for the intervention group and “No” for the comparison group. For analysis purposes, all “Yes” responses were coded as “1,” and all “No” responses were coded as “0.”; *Duration in VSLG Participation***:** Measured as the number of years of program participation, ranging from 1 to 7 years (used as a continuous reflecting its underlying ratio-scale properties and the theoretical expectation of a dose–response relationship with outcomes);

The following variables were examined as potential mediators of the association between VSLG participation/duration and depression:

*Neighborhood social capital (NSC):* NSC was used to measure experiences and feelings related to interpersonal networking using a 7-item assessment tool with a 5-point Likert scale. NSC was assessed as a composite score of seven items measuring trust, cohesion, and social interaction. The Cronbach’s α for the composite score was 0.842 [59, 60]; *Household Hunger*: Household hunger was measured using the Household Hunger Scale (HHS), which has been validated in multiple African countries. It is a composite measure, with scores ranging from 0 to 6, where 0–1 indicates little or no hunger, 2–3 indicates moderate hunger, and 4–6 indicates severe hunger [61, 62]; *Household Assets*: Household assets were measured using a 21-item asset index based on Filmer and Pritchett (1999) [57]. The index assessed material wealth, including household goods, housing quality, land, and livestock, and has demonstrated strong internal consistency (Cronbach’s α = 0.96); *Self-Rated Health (SRH):* SRH is a one-item measure reported by participants on a 4-point scale (1 = very bad to 4 = very good). SRH is widely used in epidemiological studies due to its reliability and predictive validity [63, 64].

#### Covariates

Covariates were selected a priori based on theoretical frameworks and empirical evidence linking each factor to depression outcomes. Sociodemographic characteristics, including age, sex, education, household size, marital status, years in partnership, income, and employment status, were included as established correlates of depression prevalence and severity in sub-Saharan African contexts [6, 20, 41]Spirituality was included, given evidence of its protective role against depressive symptoms in African populations, where religious and spiritual engagement often serves as a primary coping resource[65].

Additional psychosocial covariates were included to account for well-documented pathways between social functioning and depression. Perceived loneliness was controlled for given its strong and consistent association with depressive symptomatology across populations [41]. Interpersonal networking and interpersonal contact with friends were included to capture the buffering effects of social support and integration on mental health outcomes [41, 42]. Daily meal frequency was included as a proxy for food security and nutritional adequacy, both of which have been associated with depression risk in low-resource settings [41].

### Analysis

Several statistical analyses were conducted to answer the research questions. T-tests and chi-square tests were conducted to provide a summary of descriptive analyses, including frequencies and percentages for categorical variables, as well as standard deviations and intervals for continuous variables. A multi-collinearity test using the Variation Inflation Factor (VIF) was performed to rule out a significant correlation between independent variables. A VIF level of ≤ 2.5 was used to determine multicollinearity as recommended by Johnson and his colleagues [66]. Two multiple linear regression models and two SEMs were conducted for each research question. The same set of variables was used across all models. SEM enhances analytical precision by capturing complex and multiple pathways and accounting for measurement error. Both the multiple regression and SEM tests assessed the link between the two predicting factors (participation and duration in VSLG intervention) and depression, along with four predictors: household assets, NSC, general health, and household hunger. These models also included theoretically and empirically related control variables (n = 11).

## Results

### Sample characteristics

Table 1 presents the differences in sample characteristics between the total sample (n = 653), the intervention group (n = 317), and the control group (n = 336) using t-test statistical analysis. Among the total sample, 26.19% (171) had no formal education, 29.25% (191) completed primary school, 24.81% (162) completed middle school, and 19.75% (129) completed secondary school or higher. Marital status was categorized into three groups: married (57.73%, n = 377), single (18.53%, n = 121), and separated (23.74%, n = 155).

**Table 1:** Sample characteristics by VSLG participation (N = 653)

Variables	Frequency (%) or mean (SD)		
	Total sample (N = 653)	VSLG-participants (n = 317)	Non-VSLG participants (n = 336)
*Independent variables*			
VSLG participation (Yes)	317 (47.93)	317 (100.00)	0 (0.00)
Duration in VSLG (Years)***	1.50 (2.00)	3.10 (1.78)	0 (0.00)
*Dependent variables*			
Depression episodes***	12.69 (5.10)	12.10 (4.68)	13.24 (5.43)
*Mediating variables*			
Ownership of household assets***	5.03 (2.24)	5.54 (2.22)	4.56 (2.15)
Neighborhood social capital	23.51 (3.55)	23.73 (3.13)	23.31 (3.90)
General health			
Poor	25 (3.84)	8 (2.56)	17 (5.01)
Fair	221 (33.95)	95 (30.45)	126 (37.17)
Good	292 (44.85)	150 (48.08)	142 (41.89)
Very good	133 (17.36)	59 (18.91)	54 (15.93)
Household hunger***	3.04 (2.66)	2.45 (2.51)	3.60 (2.67)
*Control variables*			
Age	40.54 (12.50)	41.18 (12.32)	39.95 (12.66)
Education levels (Freq & %)***			
Not attended	171 (26.19)	80 (25.24)	91 (27.08)
Primary school	191 (29.25)	80 (25.24)	111 (33.04)
Middle school	162 (24.81)	72 (22.71)	90 (26.79)
Secondary school & above	129 (19.75)	85 (26.81)	44 (13.10)
Household size	5.83 (2.27)	5.77 (2.34)	5.88 (2.21)
Marital status ***			
Married	377 (57.73)	163 (51.42)	241 (63.69)
Single	121 (18.53)	71 (22.40)	50 (14.88)
Separated	155 (23.74)	83 (26.18)	72 (21.43)
No. of children 5 years old and younger	0.81 (0.87)	0.80 ( 0.90)	0.83 (0.84)
No. of children 6–18 years	2.20 (1.49)	2.15 (1.56)	2.25 (1.42)
No.of children above 18 years **	2.60 (1.48)	2.48 (1.61)	2.72 (1.31)
Spirituality	15.48 (5.44)	15.66 (6.24)	15.31 (4.53)
No. of meals per day***	2.58 (0.58)	2.67 (0.49)	2.50 (0.64)
Friendship network***	13.57 (5.89)	14.66 (5.21)	12.34 (6.24)
Perceived loneliness	4.65 (1.62)	4.55 (1.60)	4.74 (1.65)

*p < 0.1 ** p < 0.05, *** p < *0.01*

The t-test revealed that the intervention group indicated a statistically significantly lower mean score for depression (M = 12.10, SD = 4.68, *p* < 0.01) compared to the comparison group (M = 13.24, SD = 5.43). The study also examined four mediating variables: ownership of household assets, NSC, general health, and household hunger. The t-test results indicated that the intervention group had significantly higher means for household assets (M = 5.54, SD = 2.22, *p* < 0.01). The participant general health ordinal measure four groups: 1) Poor: 3.84% (25), 2) Fair: 33.95% (221), 3) Good: 44.85% (292), and 4) Very Good: 17.36% (133). Furthermore, the intervention group exhibited a significantly lower mean score for household hunger (M = 2.45, SD = 2.51, *p* < 0.01). However, no significant difference in NSC was found between the groups. Among the control factors, the results showed that educational level, marital status, number of children over 18 years old living in the household, number of meals per day, and interpersonal networking were statistically significant, while the remaining control variables of age, household size, perceive loneliness, and number of children under 5-years and 6–18 living in the household did not exhibit significant differences.

### Linear regression analysis

Table 2 shows results from two regression models that were used to examine the relationship between two focal predictors (women’s participation in VSLG and the duration of participation in VSLG) and depression. Model 1 presented the association between women’s depression and VSLG participation, and Table 2 reports standardized coefficients (β), standard deviations (SD), and *p*-values. In Model 1, the results indicated a significant inverse correlation between women’s participation in VSLG and depression (β = − 0.78, SD = 0.40, *p* < 0.05). This suggests that, compared to non-VSLG participants, women in the program had a lower probability of experiencing depression episodes by 0.78. NSC (β = 0.17, SD = 0.05, *p* < 0.05) and household hunger (β = 0.20, SD = 0.08, *p* < 0.001) were positively associated with depression, whereas women’s general health showed a significant negative correlation with depression (β = − 0.64, SD = 0.25, *p* < 0.001). This also suggested that an increase in household hunger (e.g., going a night without food) was associated with a 0.20 increase in depression, while a unit increase in women’s general health correlated with a 0.64 decrease in depression. However, NSC and household assets indicated a negative relationship and had no significant change on women’s depression in this model, respectively.

**Table 2:** Results of linear regression models predicting depression (N = 653)

Depression				
	Model 1		Model 2	
Independent variables	β	95% CI	β	95% CI
Participation in VSLG	− 0.78 (0.40)*	− 1.57, − 0.01		
Duration in VSLG (Years)			− 0.30 (0.10)***	− 0.50, − 0.11
*Predictors*				
Household assets	0.12 (0.09)	− 0.06, 0.31	0.15 (0.09)	− 0.03, 0.34
Neighborhood social capital	0.17 (0.05)**	0.07, 0.27	0.17 (0.05)***	0.07, 0.28
General health	− 0.64 (0.25)***	− 1.14, − 0.15	− 0.64 (0.25)***	− 1.13, − 0.15
Household hunger	0.20 (0.08)***	0.05, 0.35	0.18 (0.08)***	0.03, 0.33
*Control variables*				
Age	− 0.00 (0.02)	− 0.04, 0.03	− 0.00 (0.01)	− 0.04, 0.03
Education				
Primary school	− 0.72 (0.51)	− 1.74, 0.29	− 0.68 (0.51)	− 1.68, 0.32
Middle school	− 0.06 (0.57)	− 1.18, 1.06	0.00 (0.57)	− 1.11, 1.12
Secondary school & Above	0.79 (0.64)	− 0.48, 2.05	0.73 (0.64)	− 0.52, 2.01
Household size	0.46 (0.16)***	0.15, 0.78	0.48 (0.16)***	0.16, 0.80
Marital status				
Single	1.16 (0.51)**	0.14, 2.17	1.15 (0.51)**	0.15, 2.16
Separated	− 0.60 (0.52)	− 1.62, 0.41	− 0.56 (0.51)	− 1.57, 0.45
No. of children 5 years old and younger	− 0.52 (0.23)**	− 1.03, − 0.01	− 0.55 (0.26)**	− 1.06, − 0.46
No. of children 6–18 years old	− 0.51 (0.18)***	− 0.87, − 0.14	− 0.51 (0.18)***	− 0.87, − 0.15
No. of children above 18 years old	− 0.48 (0.18)***	− 0.83, − 0.13	− 0.48 (0.17)***	− 0.83, − 0.12
Spirituality	0.01(0.03)	− 0.06, 0.08	0.01 (0.03)	− 0.06, 0.08
No. of meals per day	− 1.01 (0.34)***	− 1.68, − 0.34	− 0.97 (0.34)***	− 1.64, − 0.30
Social net with friends	− 0.12 (0.03)***	− 0.18, − 0.05	− 0.11 (0.03)***	− 0.18, − 0.05
Percived loneliness	0.60 (0.12)***	0.35, 0.83	0.59 (0.12)***	0.36, 0.83

*p < 0.1 ** p < 0.05, *** p < *0.01*

In Model 2, the relationship between the duration of women’s participation in VSLG and depression was examined. The regression results indicated a significant inverse correlation between the duration of VSLG participation and depression (β = − 0.30, SD = 0.10, *p* < 0.001). This suggests that women who participated in the program for a longer period had a lower probability of experiencing depression episodes. Specifically, each additional year of VSLG participation was associated with a 0.30 decrease in depression. NSC (β = 0.17, SD = 0.05, *p* < 0.001) and household hunger (β = 0.18, SD = 0.08, *p* < 0.05) were positively associated with depression, while women’s general health showed a significant negative correlation with depression (β = − 0.64, SD = 0.25, *p* < 0.001). Conversely, a unit increase in women’s general health correlated with a 0.64 decrease in depression. However, household assets had no significant difference on depression in Model 2.

In both Model 1 and 2, control variables such as household size (β = 0.48, SD = 0.16, *p* < 0.001), marital status (β = 1.15, SD = 0.51, *p* < 0.05), and perceived loneliness (β = 0.59, SD = 0.12, *p* < 0.001) were positively correlated with depression. The results also indicated that single women had a higher probability of depression compared to married women (β = 1.15, SD = 0.51, *p* < 0.05). In contrast, having children under five years old (β = − 0.55, SD = 0.26, *p* < 0.05), children aged 6–18 years (β = − 0.51, SD = 0.18, *p* < 0.001), and children aged over 18 years (β = − 0.48, SD = 0.17, *p* < 0.001) living in the household, as well as the frequency of meals per day (β = − 0.97, SD = 0.34, *p* < 0.001) and social networking with friends (β = − 0.11, SD = 0.03, *p* < 0.001) were significantly negatively correlated with depression. However, demographic variables such as age, education, marital status, and spirituality did not show significant change.

### SEM 1 participation in VSLG and depression

Table 3 presents the SEM results on the relationship between women’s participation in the intervention and depression, mediated by household assets, NSC, general health, and household hunger, while controlling for all confounding variables stated above. The direct path examination between participation in the intervention and women’s depression was significantly negatively correlated (β = − 0.78, *p* < 0.05), suggesting participants of VSLGs had lower depression episodes compared to the non-VSLG participants. The direct link between participation in the intervention and the mediators showed a statistically significant positive association with household asset ownership (β = 0.74, *p* < 0.01) and general health (β = 0.14, *p* < 0.05), and an inverse correlation with household hunger (β = − 0.92, *p* < 0.01). This indicates that a one-year increase in program participation is associated with a 0.74 increase in household asset ownership and a 0.14 increase in general health, while reducing the probability of household hunger and depression episodes by 0.92 and 0.78, respectively. Duration in the intervention had no significant variation on NSC, though the direction was positive (β = 0.09, *p* > 0.05).

**Table 3:** SEM analyses of participation in VSLG to predict depression (N = 653)

Independent variable	Household assets (β)	Neighbourhood social capital (β)	General health (β)	Household hunger (β)	Depression (β)
Participation in VSLG	0.74***	0.09	0.14 **	− 0.92***	− 0.78 *
Household assets					0.12
Neighbourhood social capital (NSC)					0.17 ***
General health					− 0.64 ***
Household hunger					0.20 ***
*Control variables*					
Age	0.04 ***	0.03 **	− 0.00 **	− 0.02 *	− 0.00
*Education*					
Primary school	0.96 ***	0.07	0.08	− 0.33	− 0.72
Middle school	1.62 ***	− 0.01	0.21 **	− 1.12 ***	− 0.06
Secondary school & above	2.11 ***	0.15	0.21**	− 1.66 ***	0.78
Family size	0.13*	0.18	− 0.04 *	0.13	0.46 ***
*Marital status*					
Single	− 0.51 **	0.07	0.07	− 0.17	1.16 **
Separated	− 0.95 ***	− 0.03	− 0.03	0.47 *	− 0.60
Spiritual practices	0.01	− 0.01	− 0.01	0.02	0.01
No. of children < 5 years old and younger	− 0.28 **	− 0.17	0.08 *	− 0.06	− 0.52 **
No.of children 6 to18 years old	0.02	− 0.22	− 0.00	− 0.06	− 0.50 ***
No.of children above 18 years old	− 0.13	0.26 *	0.03	− 0.22 **	− 0.48 ***
No. of meals per day	0.26 *	0.33	0.06	− 0.84 ***	− 1.01 ***
Social net with friends	0.02	0.04	− 0.00	0.03	− 0.12 ***
Perceived loneliness	− 0.07	− 0.19**	− 0.11 ***	0.07	0.59 ***
Indirect varation	0.32***	0.00	0.35**	− 0.11	
Total change	0.51				
Model fit indices (without control)					
CFI	0.94				
TLI	0.20				
RMSEA	0.08				

*** p < 0.01, ** p < 0.05, * p < *0.1*

The path analysis between the mediating factors and depression showed a significant positive association with NSC (β = 0.17, *p* < 0.01) and household hunger (β = 0.20, *p* < 0.01), but a negative strong correlation with general health (β = − 0.64, *p* < 0.01). This suggests that a unit increase in NSC is associated with a 0.17 increase in depression episodes. This counterintuitive finding may reflect the unique circumstances of this sample. Although most literature links higher NSC with improved mental health, research suggests that this relationship does not consistently hold in marginalized or displaced populations, where strong bonding ties may increase social obligations and shared stress exposure [64, 65]. The participants in this study were largely displaced due to civil conflict and recurrent natural disasters, including cyclones and flooding, which disrupted traditional kinship networks and required rebuilding social ties under stressful conditions. In such contexts, heightened neighborhood cohesion may unintentionally intensify psychological distress through shared trauma and economic hardship, rather than buffering against it.

**Fig. 2:**
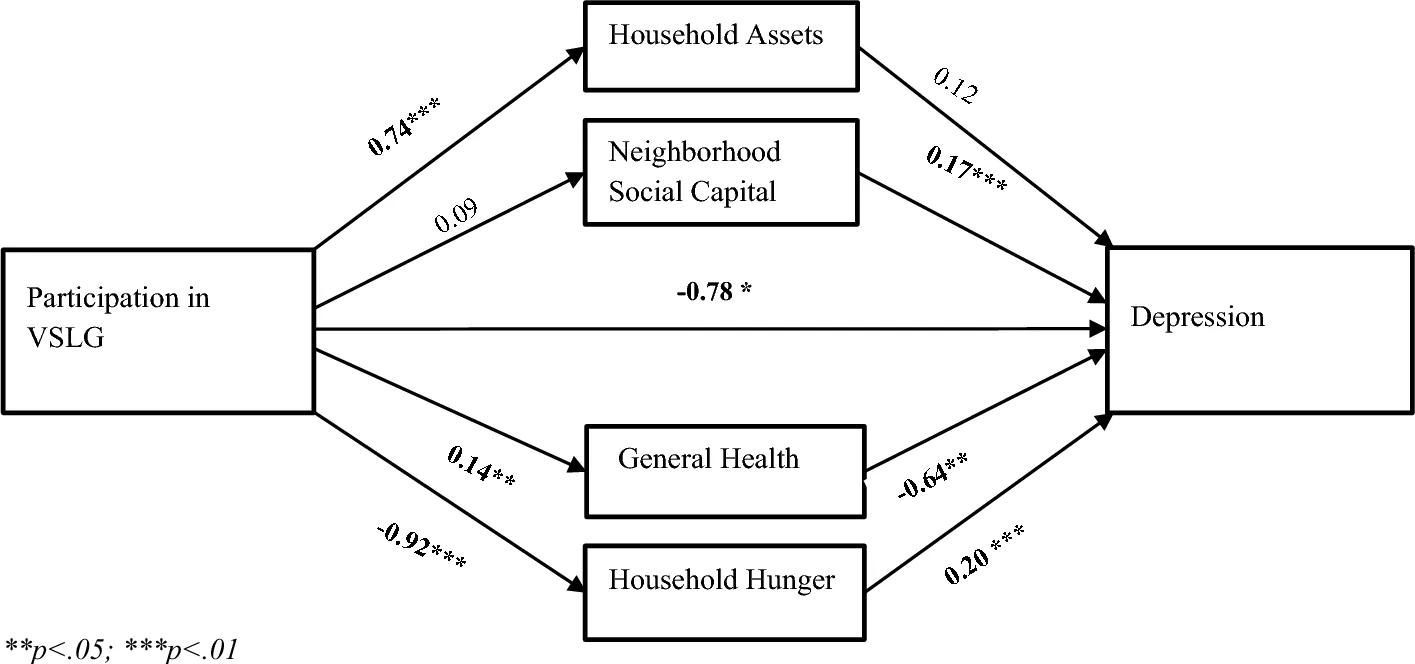
SEM analysis of participation in VSLG targeting depression indirect variation through household assets: 0.09 (*p* = 0.21); through neighborhood social capital: 0.02 (*p* = 0.66); through general health: 0.08 (*p* = 0.02); and through household hunger: 0.19 (*p* = 0.02). VSLG, village saving & loan groups

The results also indicated that an increase in household hunger (e.g., going to bed hungry) is associated with a 0.64 increase in episodes of depression. Figure 2 illustrates that, in SEM 1, participation in VSLG had both a significant direct and indirect influence on depression episodes through general health (indirect: β = 0.08, *p* < 0.01) and household hunger (indirect: β = − 0.19, *p* < 0.01). The SEM results indicated acceptable model fit regard to most of the indices, with a CFI of 0.94 and a RMSEA of 0.08. However, the TLI was below the ideal threshold. Overall, the model explained 51% of the variance in women’s quality of life (R^2^ = 0.51).

### SEM 2 duration in VSLG participation and depression

Table 4 presents the SEM results on the relationship between women’s duration in VSLG participation and depression, by household assets, NSC, general health, and household hunger, while controlling for all confounding variables included in the previous models. The direct link between the duration of participation in the intervention and women’s depression episodes was inversely significantly correlated (β = − 0.30, *p* < 0.01), suggesting that each additional year of participation in the intervention was associated with a 0.30 reduction in depressive episodes.

**Table 4:** SEM analyses of duration in VSLG to predict depression (N = 653)

Independent variable	Household assets (β)	Neighbourhood social capital (β)	General health (β)	Household hunger (β)	Depression (β)
Duration in VSLG participation	0.22 ***	0.03	0.02	− 0.25 ***	− 0.30 ***
Household assets					0.15
Neighbourhood social capital (NSC)					0.17 ***
General health					− 0.64 ***
Houshold hunger					0.18**
*Control variables*					
Age	0.04 ***	0.03 **	− 0.00 **	− 0.02 *	− 0.00
*Education*					
Primary school	0.90 ***	0.06	0.07	− 0.26	− 0.68
Middle school	1.52 ***	− 0.02	0.20 **	− 1.02 ***	0.01
Secondary school & above	2.12 ***	0.15	0.22 **	− 1.68 ***	0.73
Family size	0.13 *	0.18	− 0.04 *	0.13	0.48 ***
*Marital status*					
Single	− 0.48 **	0.50	0.08	− 0.22	1.15 **
Separated	− 0.94 ***	− 0.14	− 0.02	0.45 *	− 0.56
Spiritual practices	0.01	− 0.03	− 0.01	− 0.02	0.01
No. of children five years and younger	− 0.25 **	− 0.16	− 0.08 **	− 0.09	− 0.55**
No.of children 6 to18 Years	0.02	− 0.22	− 0.00	− 0.05	− 0.51 ***
No.of children above 18 years	− 0.13 *	− 0.26 **	0.03	− 0.21 **	− 0.48 ***
No. of meals per day	0.23	0.32	0.07	− 0.82 ***	− 0.97 **
Social net with friends	0.01	0.04	− 0.00	0.03 *	− 0.11 **
Perceived loneliness	− 0.07 ***	− 0.19**	− 0.11 ***	0.08	− 0.59 ***
Indirect varation	0.03	0.00	− 0.01	− 0.04 **	
Total change	0.52				
Model fit indices (without control)					
CFI	0.95				
TLI	0.30				
RMSEA	0.08				

*** p < 0.01, ** p < 0.05, * p < *0.1*

The direct path analysis between duration in VSLG participation and the mediators showed a statistically significant positive association with household asset ownership (β = 0.22, *p* < 0.01) and an inverse correlation with household hunger (β = − 0.25, *p* < 0.01). This indicates that a one-year increase in program participation was associated with an increase in household assets ownership by 0.22 and decrease in household hunger by 0.25. In SEM 2, duration in the intervention had no significant change on NSC and general health, though the direction of the relationship was positive.

**Fig. 3:**
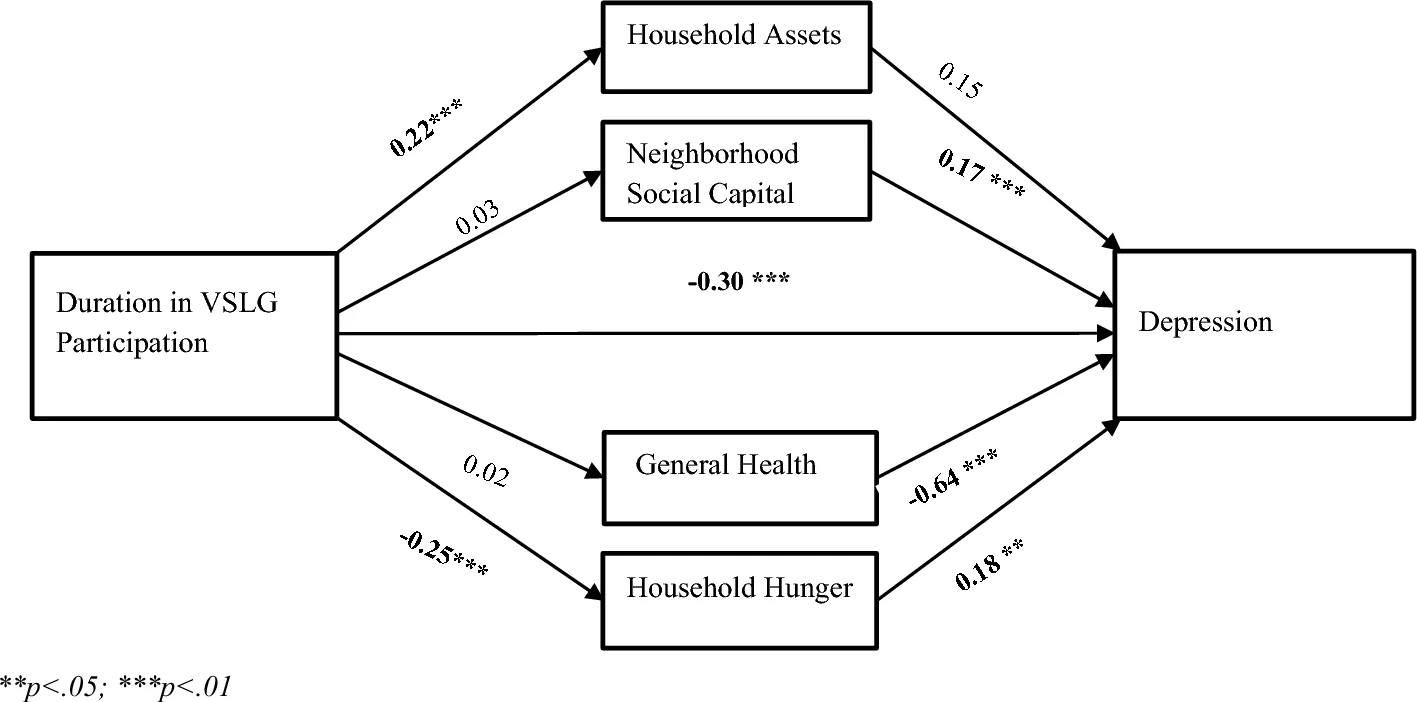
SEM Analysis of Duration in VSLG Participation Targeting Depression. Indirect variation through household assets: 0.03 (*p* = 0.12); through neighborhood social capital: 0.00 (*p* = 0.70); through general health: 0.01 (*p* = 0.17); and through household hunger: 0.04 (*p* = 0.02). VSLG, village saving & loan groups

In SEM 2, the path analysis between depression and the mediating factors showed a significant positive association with NSC (β = 0.17, *p* < 0.01), and household hunger (β = 0.18, *p* < 0.05), but a negative correlation with general health (β = − 0.64, *p* < 0.01). This also means that a unit increase in NSC is associated with a 0.17 increase in episodes of depression, which is not consistent with most of existing evidence. The results also indicated that an increase in household hunger is associated with a 0.18 increase in episodes of depression. However, an inverse correlation with women’s general condition suggested that a unit increase in general health correlated with a lower depression score by 0.64. Figure 3 illustrates that, in SEM 2, duration in VSLG participation had a significant direct and indirect impact on depression through household hunger (indirect: β = 0.04, *p* < 0.05). The SEM results indicated acceptable model fit for most of the indices, with a CFI of 0.90 and an RMSEA of 0.08. However, the TLI was below the ideal threshold. Overall, the model explained 52% of the variance in women’s depression (R^2^ = 0.52).

As the SEM models indicated, the TLI fell below the recommended threshold. Several factors can influence the TLI and contribute to low TLI, including small sample size, model complexity, and inadequate representation of theoretical constructs [67]. The inclusion of numerous control variables, such as a large number of indicator variables, some of which are categorical, likely contributed to the decline in the average estimated TLI [68, 69]. Previous studies have also emphasized that failure to capture complex relationships in the data [70], along with skewed variables, can further lower the TLI [71].

## Discussion

Overall, the study findings support the premise that FC strategies are recommended and widely recognized as a key pillar of the United Nations’ 2030 Sustainable Development Goals (SDGs). These strategies are actively promoted by global institutions such as the World Bank and the UN to advance financial stability, foster human development, and improve life outcomes [72, 73]. Notably, FC initiatives have been implemented in more than 40 African countries, including Mozambique, as part of broader efforts to combat poverty and stimulate both human and economic development. These mechanisms are particularly impactful for low-income populations, especially women, who often face systemic barriers to financial access. By expanding opportunities for financial access, FC interventions contribute to both subjective and collective improvements in well-being, enhancing the financial and non-financial dimensions of livelihoods [74].

The regression model results revealed depression was negatively and significantly correlated with VSLG participation and duration of VSLG participation, as well as with other confounders in the model, including children of all ages living in the household, frequency of daily meals, and interpersonal networking with friends. Similarly, the SEM examinations showed a significant inverse direct influence of both participation in VSLG and duration in the program on women’s depression episodes.

The SEM path examination of these models revealed a significant role of participation in VSLG in increasing the probability of household asset ownership, improving general health, and availability of food, with a non-significant positive association with NSC. The path examination from the mediators to depression also indicated a significant positive and negative link with women’s general health and household hunger, respectively. Furthermore, the models revealed a strong indirect influence of the VSLG intervention on depression episodes through improved general health conditions and increased the probability of food availability in the household (i.e., consistent with previous evidence from Mali and Zanzibar [74, 75]. Among the demographic and other theoretically linked confounding variables, family size, frequency of meals, and perceived feelings of loneliness were strongly linked with women’s depression episodes.

In line with the vast majority of existing evidence, this study’s findings also support the assumption of the linear economic development stage theory, which proposes that financial products and resources, such as the accumulation of savings, assets, and investment, advance improvements in mental health [76, 77]. The results of this study align with evidence from several sub-Saharan African (SSA) countries, including Ghana, Nigeria, Ethiopia, Malawi, and South Africa, which highlights the critical role of socioeconomic and demographic factors, as well as the importance of meeting basic needs, in shaping key health determinants such as interpersonal relationships and economic well-being [72, 78].

As previous studies widely suggested, women are twice as likely to experience depression compared to men due to different biological, socioeconomic, cultural, and cognitive factors, including the disproportional wealth gap [79]. These factors are more critical in Africa’s rural agricultural settings, where resources and infrastructural barriers, coupled with the cultural and biological burdens women face daily in the household and farm settings, further exacerbate the issue. In such a reality, in addition to the financial benefits (accumulation of savings over time and building assets), participation in VSLG facilitates social interactions, trust, reciprocity, and experience sharing, which also potentially creates an emotional home, helps with seeking support, making better decisions, and solving problems as participants consistently meet together [80, 81].

This study’s findings about NSC aligned with previous findings regarding the gap among various social capital assumptions. For example, the neighborhood resource social capital theory lacks consistency in addressing the complexity of health factors across different socioeconomic and demographic settings, particularly in a micro setting [82, 83]. Nevertheless, studies also suggest that [84] that in environments where homogeneity and bonding are maintained through social cohesion, trust, and affiliation at both the individual and community levels, the role of neighborhood social capital is more vital[85].

### Limitations

Despite the valuable contributions of this study in exploring a self-help, community-based approach that promotes both social and financial benefits to improve mental health among low-income households, several limitations must be acknowledged. First, the cross-sectional post-intervention design limits causal inference; a randomized experimental design would have provided stronger evidence of the effects of VSLG participation on depression. Second, the study’s sample was limited to rural low-income communities in the Beira region of Central Mozambique, which may affect the generalizability of the findings to broader or urban populations. Third, recall bias may have influenced responses, as participants were asked to remember events over varying time frames, such as depression in the past seven days or financial events over the past two years. Fourth, social desirability bias could have affected responses, although this risk was minimized by using trained external enumerators. Fifth, as previously discussed, the Tucker–Lewis Index (TLI) model fit criteria across the SEM models fell below the recommended threshold. The interpretation of these findings should be interpreted with consideration of the relatively poor model fit, which may limit the precision and generalizability of the estimates. However, the researchers chose to include all existing variables and confounders that are theoretically and empirically associated, including potential socioeconomic and demographic factors, which may have further influenced the model fit. Sixth, although the duration of VSLG participation spanned a relatively narrow range (1–7 years), treating it as continuous preserved statistical efficiency and avoided the arbitrary thresholding inherent in categorization. Finally, the study sample had a gender imbalance, with fewer male participants, resulting in a primary focus on women in both the literature and analysis. Nonetheless, the study offers important academic and practical implications for financial capability interventions to improve the quality of life and life outcomes among underserved populations.

### Implications & recommendations

This study’s FC approach promotes the role of community-based FC approaches in addressing disparities in mental health in influencing key markers of poverty, such as financial products (asset building), interpersonal skills and behaviors, general health, and food security [86]. These outcomes align with various Sustainable Development Goals (SDGs. For instance, findings showed that VSLG participants reported significantly better general health, and that improved health mediated the relationship between participation and reduced depression, which aligns with SDG 3 (Good Health and Well-Being). VSLGs facilitate access to funds for healthcare and emergencies, encourage informed health-related financial decisions, and reduce stress by improving affordability and purchasing powers. These practices improve psychosocial well-being and overall health. Linking financial interventions with health programs offers actionable strategies for enhancing community health outcomes.

In this study, VSLG participation was associated with significantly higher household asset ownership and reduced household hunger, demonstrating concrete pathways to poverty reduction. These results suggest that FC interventions are a critical alternative for Mozambique women to ease the burden of poverty and its complex outcomes that put the population with an estimated annual income of $1.228 per capita and 46% living at risk for depression [87–90]. These findings align with SDG 1 (No Poverty). Other studies have also suggested the anti-poverty benefits of access to other community-based FC interventions, such as microfinance cooperative practices, for coping with seasonal agricultural shocks and food prices [20].

Rural women are often excluded from development gains, yet their empowerment is vital to break the poverty cycle and advance rural development aligned with SDG 5 (Gender Equality). This study focused on women’s financial inclusion, which promoted empowerment and gender equality. Participation in VSLGs enhances women’s access to financial resources and supports positive financial behaviors, including independent saving, investment, and active participation in household financial decision-making. These changes contributed to improved financial outcomes, such as increased income generation, asset ownership, and strengthened economic independence. Collectively, these outcomes enhance women’s decision-making power, social participation, and self-confidence, supporting broader efforts to advance gender equality and women’s socioeconomic empowerment. These findings align with the WET that advances women’s agency by addressing empowerment and equity barriers that hinder women’s advancement [27]. Based on the work of Naila [28], WET emphasizes access to resources and participation in decision-making, which enhances women’s ability to act independently and make strategic life choices.

Amartya Sen’s Capability Approach, which defines development as the expansion of people’s real freedoms and capabilities to achieve valued ways of being and doing, also align with WET [23, 25, 91]. As it is indicated in these studies, the approach implies that economic resources matter only when women can convert them into genuine capabilities, such as control over earnings, mobility, voice in household decisions, and participation in local governance. In rural contexts, where social norms and structural barriers limit this conversion, integrated capacity development and informal financial models like the VSLGs enhance financial freedom, collective agency, and bargaining power. This linkage between financial capability and expanded freedoms creates a concrete pathway for rural women’s substantive participation in public decision-making and governance.

Drawing on the results of this study, we suggest four key recommendations: first, governments in SSA, especially Mozambique, should integrate the community-based financial capability intervention of VSLGs into rural development policies. Such policies should include the model studied here, which supported women organizing themselves into VSLGs, rather than being organized and administered by an NGO. Second, the policies should facilitate the ability of NGOs and community leaders to promote sustainable, locally led financial initiatives such as VSLGs, reducing dependence on donor-driven models and providing seed money [92], while building financial literacy, trust, and social cohesion. The VSLG approach enhances financial access through savings and credit opportunities, promotes financial behaviors, and improves household income and assets. These outcomes reduce poverty and strengthen economic resilience. Policy support for community-based savings groups can further expand financial inclusion for vulnerable populations. Third, research is needed on the long-term impact and adaptability of community-based FC strategies in rural SSA settings. Longitudinal and cross-regional studies should explore how context influences outcomes related to poverty, quality of life, and women’s empowerment [78], with attention to gender, education, and other social factors [93]. Finally, further research should examine how VSLGs build neighborhood social capital and improve mental health and life outcomes. Mixed-methods designs will enrich findings and support more informed policy and practice [94].

## Data Availability

The datasets generated and/or analyzed during the current study are not publicly available due to an embargo and data ownership of the agency who implemented the intervention and granted permission to the first author. However, the data may be made available on reasonable requests and with the agreement of both the first author and the agency once the restriction(embargo) period has ended.

## Declarations

### Clinical trial registration

Not applicable; this study was not a clinical trial.
